# Super enhancers as key drivers of gene regulatory networks in normal and malignant hematopoiesis

**DOI:** 10.3389/fcell.2025.1674470

**Published:** 2025-11-14

**Authors:** Ying Cheng, Guangxin Pei, Hengchao Zhang, Yan Hou, Lei Sun, Hongdi Xu, Yuning Lv, Xiuyun Wu

**Affiliations:** School of Life Sciences, Zhengzhou University, Zhengzhou, China

**Keywords:** super enhancers, gene expression regulation, hematopoiesis, hematologicalmalignancies, oncogene regulation

## Abstract

Super enhancers (SEs) are clusters of enhancers with exceptionally high transcriptional activity, crucial for determining cell identity and regulating gene expression. They function as key regulatory hubs, governing gene networks essential for normal hematopoiesis while also driving the pathogenesis of hematological malignancies. This review summarizes the role of SEs in maintaining hematopoietic lineage identity and examines how their dysregulation in acute myeloid leukemia (AML), myelodysplastic neoplasms (MDS), adult T-cell leukemia (ATL), acute lymphoblastic leukemia (ALL), and multiple myeloma (MM) leads to oncogenic activation. By regulating key oncogenes, SEs represent promising therapeutic targets. Emerging strategies-such as BET inhibitors, CDK7/9 inhibitors, and rational drug combinations-effectively disrupt SE-driven transcriptional programs and show potential to overcome treatment resistance in these cancers.

## 1 Introduction

Eukaryotic transcription is a tightly regulated process, dependent on coordinated interactions between transcription factors, co-regulators, and cis-acting DNA elements, including enhancers, promoters, and silencers. Enhancers were first identified in 1981 when Banerji et al. discovered a class of short DNA sequences capable of activating transcription in a manner independent of their position, distance, or orientation relative to gene promoters (Banerji et al., 1981). Beyond enhancers’ classical role as transcription factor platforms, enhancers are actively transcribed into non-coding RNAs. These enhancer-derived RNAs (eRNAs) cooperate with architectural proteins to stabilize chromatin looping configurations, thereby potentiating enhancer-promoter communication for transcriptional activation (Orom et al., 2010; Wang et al., 2025; Akiki et al., 2024). Super enhancers (SEs) are large clusters of transcription enhancers that play a crucial role in regulating genes necessary for cellular identity and various biological processes (Hnisz et al., 2013). Distinguished from typical enhancers, SEs amplify transcriptional output, including abundant eRNAs, and enrich histone modifications (e.g., H3K27ac), thereby driving high-level gene expression in developmental and disease contexts (Khan et al., 2018; Blayney et al., 2023; Shin, 2018). Given the powerful regulatory role of SEs in gene expression, research on SEs and diseases in recent years has mainly focused on malignant tumors, indicating that they play important regulatory roles in important biological processes such as malignant tumor occurrence, cell differentiation, and immune response (Wang et al., 2023; van Groningen et al., 2017; Kai et al., 2021; Higashijima and Kanki, 2022).

Hematopoiesis is a precisely regulated developmental process whereby multipotent hematopoietic stem cells (HSCs) in the bone marrow microenvironment undergo progressive lineage commitment to generate all mature blood cell types, including erythrocytes, leukocytes, and platelets. Recent evidence increasingly highlights the important role of SEs in hematopoietic cell commitment and differentiation by facilitating dense transcription factor binding and sustaining high-level gene expression of key regulatory genes (Walker et al., 2023; Xie and Dean, 2025; Alvarez-Dominguez et al., 2017). Malignant hematopoiesis frequently arises from the aberrant activation of oncogenic pathways, wherein dysregulated expression or mutation of key oncogenes disrupts normal hematopoietic differentiation and promotes uncontrolled proliferation. Accumulating evidence demonstrates that SEs play pivotal roles in the initiation and progression of malignant hematopoiesis by dysregulating key oncogenic transcriptional programs (Jia et al., 2019; Fang et al., 2022; Wan et al., 2024). Given the critical role of SEs in defining cellular identity and driving oncogene expression in hematopoiesis, elucidating their molecular mechanisms may yield novel therapeutic approaches for hematological malignancies.

In this review, we systematically summarize recent advances in SEs research within the hematopoietic system, focusing on their role in activating gene expression during both normal and malignant hematopoiesis. Moreover, we critically evaluate emerging therapeutic strategies that target SEs regulatory networks in hematological malignancies, providing a conceptual framework for the development of novel targeted therapies against hematological disorders.

## 2 The characteristics and definition of super enhancers

**FIGURE 1:**
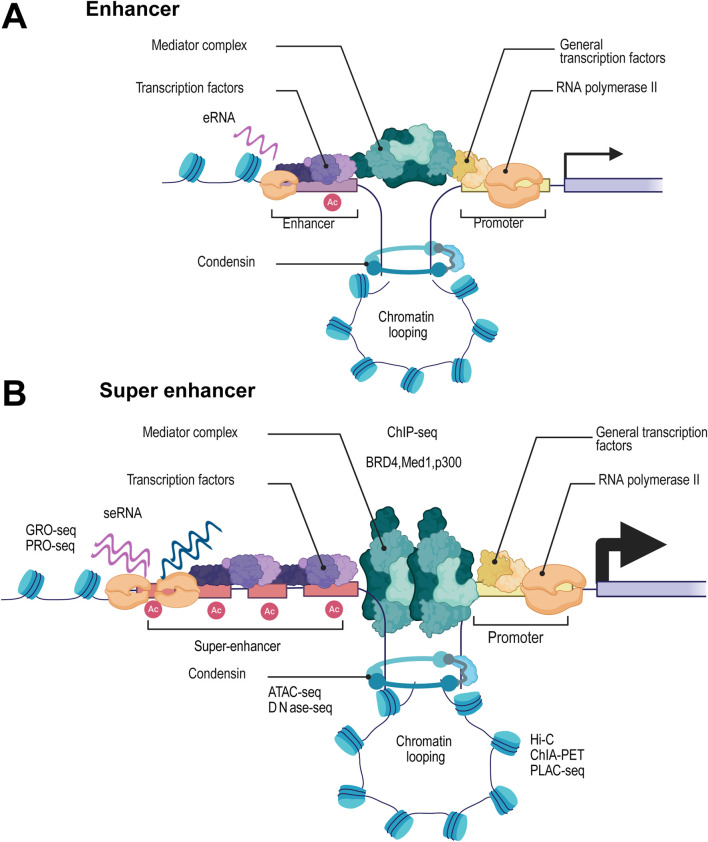
Structural and Functional Characteristics of Super Enhancers and Their Regulatory Role in Transcription Compared to Traditional Enhancers. Schematic diagram showing the structural and functional characteristics of traditional enhancers **(A)** compared with super enhancers (SEs) **(B)**. SEs are characterized by their extensive genomic span and high enrichment of regulatory elements. Compared with traditional enhancers, SEs exhibit significantly higher activity and complexity, which is reflected in the dense accumulation of histone modifications such as H3K27ac (marked as “Ac”) and H3K4me1. Additionally, SEs show strong enrichment of RNA polymerase II, facilitating robust transcriptional activity and the generation of super enhancer-derived RNAs (seRNAs). These features collectively enable SEs to exert a more potent regulatory role in gene expression compared to traditional enhancers.

In 2013, the concept of SEs was first proposed as a cluster of gene motifs containing multiple enhancers. Through ChIP-seq analysis of transcription factors and histone modification markers like H3K27ac and H3K4me1, SEs were characterized by their extensive genomic span and high enrichment of regulatory elements, significantly surpassing traditional enhancers in activity and complexity (Whyte et al., 2013; Lovén et al., 2013), as shown in Figure 1, compared to traditional enhancers, SE regions exhibit dense enrichment of histone modifications such as H3K27ac and H3K4me1, as well as strong accumulation of RNA polymerase II(RNAP II), thereby promoting efficient transcriptional activity and generating super enhancer-derived RNAs (seRNAs). Enhancer RNAs (eRNAs), a class of long non-coding RNAs (lncRNAs), are typically transcribed unidirectionally from enhancer regions, which can recruit transcription factors and promote the enrichment of H3K27ac at these sites, also as a well-established marker of active enhancers (Kim et al., 2010; Hsieh et al., 2014; Mousavi et al., 2013; Schaukowitch et al., 2014). Not surprisingly, SEs accumulate more RNAP II and are more highly transcribed, generating a large amount of long non-coding RNAs, known as seRNAs (Alvarez-Dominguez et al., 2017). Unlike classical eRNAs, seRNAs are transcribed bidirectionally from SEs regions and exist in various lengths with different functions. Among them, non-polyadenylated short seRNAs mainly exert cis-regulatory functions, while polyadenylated seRNAs in the nucleus, which are more stable, can participate in trans-regulatory roles (He et al., 2025; Font-Tello et al., 2020).

Given the unique features of SEs, high-throughput sequencing techniques have become essential tools for SE research and identification. Traditional approaches, particularly those analyzing histone modifications, predominantly use ChIP-seq technology. This method enables precise SE localization by detecting the enrichment of specific histone marks, such as H3K27ac and H3K4me1 (Font-Tello et al., 2020; Kang et al., 2021) Moreover, SE regions exhibit strong enrichment of transcription factors and coactivators, including MED1, BRD4, and p300. Combining ChIP-seq data from multiple factors allows for even more accurate SE prediction (Cai et al., 2024; Zheng et al., 2022; Liu et al., 2022). SEs are usually located in highly open chromatin regions. High-throughput sequencing techniques for checking chromatin accessibility, such as ATAC-seq and DNase-seq, can further verify the presence of SEs (Hnisz et al., 2013; Zhou et al., 2025). SE regions display enhanced bidirectional transcription, typically associated with more active enhancer RNA (eRNA) synthesis. Techniques such as GRO-seq and PRO-seq can directly capture these nascent eRNAs and the pronounced bidirectional transcription signals, enabling precise identification of SE regions (Wang et al., 2025). Furthermore, SE frequently interacts with multiple target gene promoters through chromatin loops. Hi-C technology enables genome-wide analysis of chromatin 3D structure, quantifying interaction frequencies between SEs and their target gene promoters (Huang et al., 2018). When integrated with methods like ChIA-PET and PLAC-seq, these approaches provide deeper insights into SE-mediated regulatory networks, allowing for more accurate functional characterization of SEs (Cao et al., 2017; Nott et al., 2019). These multidimensional approaches enable not only precise mapping of SEs but also uncover their central role in gene regulation.

## 3 SE-mediated gene activation orchestrates hematopoiesis

Hematopoiesis is the process by which various blood cells develop and mature in hematopoietic organs or sites. HSCs possess the dual capacity for self-renewal and multilineage differentiation, enabling them to generate all lineages of mature blood cells. In the process of differentiation from HSCs to mature blood cells, it goes through the stages of multipotent and directed hematopoietic progenitor cells, ultimately differentiating into all series of mature blood cells, including red blood cells, megakaryocytes (platelets), macrophages, eosinophils, basophils, neutrophils, T cells, B cells, and natural killer cells (Pouzolles et al., 2016; Wei et al., 2020). Studies have demonstrated that genes associated with SEs exhibit more pronounced expression changes during hematopoietic cell differentiation compared to those regulated by typical enhancers (Huang et al., 2016). Here, we review recent studies to elaborate in detail on elucidating the crucial role of SEs in driving gene activation that governs the function of hematopoietic stem and progenitor cells, lineage differentiation, as well as cell fate inheritance.

### 3.1 SEs maintain HSCs function by activating the expression of lineage-specific genes

SEs critically regulate HSCs fate by fine-tuning gene expression to ensure proper lineage output. For instance, an evolutionarily conserved SE located distally from MYC is essential for its expression in both normal and leukemic HSCs in mice and humans. Deletion of this enhancer leads to loss of c-MYC expression, resulting in differentiation defects and loss of myeloid and B-cell lineages-a phenotype resembling that of conditional MYC knockout (Bahr et al., 2018). Similarly, the RUNX1 intronic enhancer (eR1), embedded within a large HSC-specific SE, serves as a key hub where RUNX1 cooperates with factors such as TAL1, GATA2, and PU.1, making it a pivotal regulatory element in HSCs biology (Liau et al., 2017; Ng et al., 2010). Additionally, NFIX has been shown to co-localize with other transcription factors at SEs to support progenitor differentiation and homeostasis (Walker et al., 2023). Together, these studies underscore that SE-mediated transcriptional activation is fundamental to HSCs maintenance, lineage allocation, and functional integrity.

### 3.2 SEs dynamically regulate macrophage inflammation and polarization through transcriptional and epigenetic mechanisms

In macrophages, inflammatory signal transduction responds to external stimuli, triggering immediate and drastic changes in gene transcription levels (Ghisletti et al., 2010). SEs play a crucial role in regulating gene expression in macrophages, particularly those involved in immune responses and inflammation. They achieve this goal by recruiting transcription factors, dynamic eRNA transcription, and epigenetic regulation. In macrophages, Toll-like receptor 4 (TLR4) signaling dynamically reorganizes SE activity, inducing enhancer RNA (eRNA) transcription at activated immune genes while suppressing it at repressed loci (Hah et al., 2015). This SE landscape can be reshaped by metabolic stimuli such as palmitic acid, which activates inflammatory SEs and suppresses those associated with phagocytosis (Tanwar et al., 2025). The epigenetic reader ZMYND8 further fine-tunes macrophage inflammation by partnering with NF-κB/p65 to silence specific SEs (Jia et al., 2021). Furthermore, SE integrity is essential for macrophage polarization. BRD4 inhibition disrupts SE-driven expression of polarization master regulators like IRF4, impairing both M1 and M2 programs in alveolar macrophages (Li et al., 2025). Similarly, BRD4/P300-dependent SEs facilitate M2 polarization during L. donovani infection (Das et al., 2021). Targeting SE mechanisms thus offers promising therapeutic avenues for inflammatory and macrophage-related diseases.

### 3.3 SEs orchestrate erythroid development and globin gene expression

SEs are master regulators of erythroid-specific gene expression, coordinating transcriptional programs essential for red blood cell formation. Although H3K27ac marking and SE activity generally decrease during erythropoiesis, SEs remain critical for lineage commitment (Romano et al., 2020). Functional studies highlight key SE-derived components: the long non-coding RNA lncRNA-EC7/Bloodlinc promotes terminal maturation (Alvarez-Dominguez et al., 2017); The SLC25A37 SE exhibits a tiered architecture ensuring precise gene control (Huang et al., 2016); and CPOX-eRNA, transcribed from a SE spanning the Cpox locus, drives erythroblast proliferation and enucleation (Xie and Dean, 2025). Beyond the essential role of Epo in erythropoiesis, genome-wide mapping has revealed Epo-responsive SEs that regulate key erythroid factors like TAL1, establishing SEs as master regulators of the transcriptional circuitry governing erythroid maturation (Hay et al., 2016).

SEs play an essential role in ensuring balanced expression of globin genes, which is critical for hemoglobin synthesis and erythrocyte function. Studies on the α-globin locus demonstrate that its SEs function autonomously yet cumulatively to control transcriptional output and chromatin architecture (Hay et al., 2016). Disruption of individual enhancers leads to α-globin downregulation and embryonic lethality in mice (Blayney et al., 2023), while inversion of the SE results in α-thalassemia-like phenotypes (Kassouf et al., 2025). Similarly, at the β-globin locus, SE-derived seRNA facilitates RNA polymerase II (Pol II) recruitment to promote β-globin expression (Gurumurthy et al., 2021). Targeting the SE-mediated regulation of globin expression could provide a promising therapeutic approach for hemoglobinopathies such as thalassemia and sickle cell anemia, potentially opening new avenues for clinical intervention.

### 3.4 SEs serve as transcriptional hubs that orchestrate lymphocyte fate

SEs serve as transcriptional hubs that orchestrate lymphocyte fate by integrating diverse signals to control the development, differentiation, and immune function of B cells, T cells, and NK cells. In B cells, SEs function as critical platforms for NF-κB signaling, which drives chromatin opening and threshold gene expression during B cell receptor (BCR) activation (Basso and Dalla-Favera, 2015; Michida et al., 2020). Beyond NF-κB, SEs recruit factors like PU.1 and are hijacked by Epstein-Barr virus (EBV) to utilize STAT5/NFAT for survival (Wibisana et al., 2022; Zhou et al., 2015). SEs also directly regulate the immunoglobulin heavy chain (IgH) locus, enabling VDJ recombination and antibody production (Saintamand et al., 2017). This multifaceted regulation establishes SEs as master integrators of B cell identity and adaptive immune responses.

SEs in T cells are enriched for single nucleotide polymorphisms linked to immune-mediated diseases, highlighting their significance in genetic predisposition (Witte et al., 2015; Vahedi et al., 2015). Studies have shown that the transcription regulator Aire binds to and activates SEs, and topoisomerase 1 is the main Aire partner co localized on SEs and necessary for Aire to interact with all its other binding partners (Bansal et al., 2017). Recent research has shown that B4galt5 within an SE is essential for NK cell survival and the expansion of cytotoxic CD8^+^ T cells (Morrison et al., 2025). Together, these findings position SEs as central nodes integrating genetic, epigenetic, and functional axes of T cell biology, with broad implications for understanding immune pathogenesis and therapeutic targeting.

**FIGURE 2:**
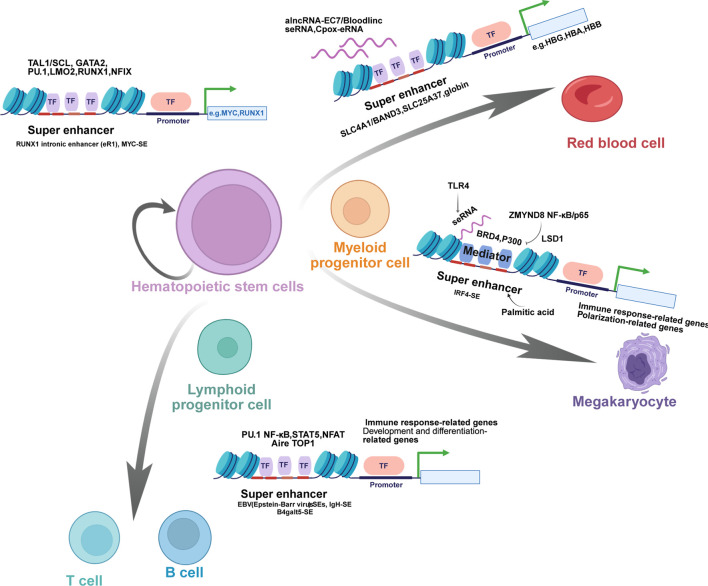
Super Enhancers in the Regulation of Hematopoietic Stem Cell Differentiation and Lineage-Specific Gene Expression. Schematic diagram illustrates the critical role of super enhancers (SEs) in governing the differentiation of hematopoietic stem cells (HSCs) into various lineages of mature blood cells and regulating lineage-specific gene expression. HSCs, with the capacity for self-renewal and multilineage differentiation, give rise to multipotent and lineage-committed progenitor cells, including myeloid progenitor cells, lymphoid progenitor cells, and megakaryocytes, which further differentiate into specialized cell types such as red blood cells, T cells, B cells, and natural killer (NK) cells. SEs mediate this process by targeting key genes and recruiting specific transcription factors (TFs). For instance, SEs such as the RUNX1 intronic enhancer (eR1) and MYC-SE regulate the self-renewal and differentiation of HSCs through interactions with TFs like TAL1/SCL, GATA2, PU.1, LMO2, RUNX1, and NFIX. In red blood cells, SE-derived RNAs (e.g., alncRNA-EC7/Bloodlinc seRNA, Cpox-eRNA) and SEs controlling globin genes (e.g., HBG, HBA, HBB) are involved in terminal differentiation. For lymphoid lineages, SEs such as EBV SEs, IgH-SE, and B4galt5-SE coordinate the development and function of T cells and B cells, with enrichment of TFs including PU.1, NF-κB, STAT5, NFAT, Aire, and TOP1. Collectively, these SE-mediated regulatory networks ensure precise lineage commitment and functional specialization during hematopoiesis, highlighting their pivotal role in maintaining hematopoietic homeostasis.

SEs serve as pivotal transcriptional hubs that govern cell identity and function across hematopoietic lineages. As we have shown in Figure 2, SEs are essential for maintaining HSPCs by activating key genes like MYC and RUNX1, and SEs dynamically regulate inflammation and polarization of macrophages by integrating external signals, undergoing epigenetic remodeling, and controlling master regulators. During erythroid development, SEs orchestrate maturation, proliferation, and globin gene expression, Furthermore, SEs integrate diverse signals to direct lymphocyte fate in B cells, T cells, and NK cells, thereby regulating adaptive immunity and being implicated in immune pathologies. Collectively, these findings underscore the fundamental role of SE-mediated transcriptional mechanisms in hematopoiesis and immunity.

## 4 SEs drive oncogene transcription in hematological malignancies

SEs play a pivotal role in the transcriptional regulation of critical oncogenes, including MYC, by orchestrating the formation of high-density transcriptional complexes that drive aberrant gene expression. In various hematological malignancies including leukemia, lymphoma, myelodysplastic neoplasms (MDS) and multiple myeloma (MM), SEs-mediated dysregulation of key oncogenes has been mechanistically established as a critical driver of tumorigenesis through multiple interconnected pathways. Firstly, SEs facilitate long-range genomic interactions by forming chromatin loops that bridge distal regulatory elements with target gene promoters, dramatically amplifying oncogenic transcription; SEs coalesce transcriptional coactivators and RNA polymerase II into high-density nuclear hubs, creating a permissive environment for runaway gene expression; additionally malignancy-defining transcription factors exhibit preferential binding to SEs, where they nucleate the assembly of oncogenic transcriptional complexes that override normal regulatory checkpoints (Debek and Juszczynski, 2022; Belloucif and Lobry, 2022). This SE-driven transcriptional addiction not only sustains hyperproliferative signaling but also underlies acquired resistance to conventional therapies, positioning SEs as high-priority epigenetic targets for next-generation precision oncology interventions.

### 4.1 SEs driven oncogene activation in AML

Acute myeloid leukemia (AML) represents an aggressive hematological malignancy originating from the malignant transformation of myeloid progenitor cells, comprising 7.6% of all hematopoietic system cancers. This disease is characterized by uncontrolled proliferation of immature myeloblasts in the bone marrow, leading to bone marrow failure and impaired hematopoiesis. Current therapeutic challenges are reflected in the dismal 5-year survival rate of <30%, highlighting the urgent need for novel treatment strategies (Kayser and Levis, 2023; Récher, 2021; Pasquer et al., 2021). Dysregulation of key leukemogenic drivers, such as MYC, HOXA, and MEIS1, triggers a cascade of carcinogenic events in AML (Luo et al., 2005; Xie et al., 2025; Zhou and Lu, 2023), and SEs play a central role in these dysregulation. In 2013, a lineage-specific SE cluster spanning 1.7 Mb downstream of the MYC transcription start site was identified at the MYC locus. These distal regulatory elements harbor focal DNA repeats that are essential for MYC expression. Importantly, these SEs depend on the chromatin remodeler BRG1 (*SMARCA4*) to sustain the MYC oncogenic transcriptional program, thereby driving AML progression (Shi et al., 2013). Integrative genetic, genomic, and biochemical analyses have demonstrated that the MYC SE region is co-occupied by BRD4, the histone acetyltransferase p300/CBP, and key hematopoietic transcription factors (including PU.1, FLI1, and MYB). This coordinated assembly forms a chromatin-based signaling hub that drives oncogenic transcription and sustains leukemia maintenance in AML (Roe et al., 2015). Studies have demonstrated that chromosomal rearrangements involving inv (3) (q21q26.2) or t (3; 3) (q21; q26.2) in AML induce leukemogenesis through dual dysregulation of GATA2 and EVI1. These structural alterations generate a SE at the EVI1 locus, driving its aberrant overexpression while simultaneously causing haploinsufficient GATA2 expression - a pathogenic combination that promotes leukemic transformation. (Gröschel et al., 2014). Ottema et al. found that a translocation of a MYC super enhancer (MYC SE) to the EVI1 locus, therefore overexpression of EVI1 in AMLs harboring a t (3; 8) (q26; q24) (Ottema et al., 2021). Mechanistic studies have revealed that the t (10; 17) (p15; q21) chromosomal translocation generates an oncogenic *ZMYND11-MBTD1* fusion protein. This chimeric protein recruits the NuA4/Tip60 histone acetyltransferase complex to SEs of key leukemogenic drivers (including HOXA, MEIS1, MYB, MYC, and SOX4), thereby establishing a pro-leukemic transcriptional program that promotes malignant transformation (Li et al., 2021). Another researcher highlighted that TRIB1 orchestrates the eviction of C/EBPα specifically from SEs, unleashing a HOXA9-dependent oncogenic cascade comprising ERG, SPNS2, RGL1, and PIK3CD, which drives leukemogenesis in AML (Yoshino et al., 2021). These studies converge to reveal SEs as critical epigenetic nodes in AML, hijacked by diverse genetic lesions to activate oncogenic networks that fuel leukemogenesis.

### 4.2 SEs silencing as a sore pathogenic mechanism in MDS

MDS formerly known as myelodysplastic syndromes, are clonal hematopoietic malignancies that cause morphologic bone marrow dysplasia along with anemia, neutropenia, or thrombocytopenia. MDS are associated with an increased risk of AML. The yearly incidence of MDS is approximately 4 per 100 000 people in the United States and is higher among patients with advanced age (Sekeres and Taylor, 2022). In AML with MLL gene rearrangements, leukaemia stem cells exhibit unique gene expression patterns and chromatin states, which are thought to function as enhancers. Concurrently, enhancer dysfunction is commonly observed in leukemia stem cells (Pan et al., 2023). Lysine-specific demethylase 1 (LSD1) is a critical epigenetic regulator in myeloid malignancies, including MDS that progress to overt leukemia. The novel LSD1 inhibitor NCD38 targets MDS-related leukemic cells by derepressing abnormally silenced SEs of hematopoietic differentiation genes. Specifically, NCD38 elevates H3K27ac levels on SEs, activating ∼500 new SEs and a core set of 62 LSD1-suppressed genes (including key regulators GFI1 and ERG). SE activation precedes transcriptional upregulation and drives myeloid differentiation programs, while simultaneously disrupting leukemogenic pathways. Depletion of SE-driven GFI1 attenuates differentiation, confirming the functional link. Crucially, SE reactivation by NCD38 eradicates primary MDS-derived leukemia cells with complex karyotypes *in vivo*, demonstrating that LSD1-mediated SE silencing sustains MDS leukemogenesis, and its reversal offers a potent therapeutic strategy against high-risk MDS (Sugino et al., 2017).

### 4.3 SEs as central orchestrators of oncogenic transcription in ATL

Adult T-cell leukemia/lymphoma (ATL) is a T-cell lymphoma caused by human T-cell leukemia virus type I (Cook et al., 2019). Controlling gene expression through splice enhancers, or SEs, plays a vital role in the regulation of alternative splicing events, which are essential in the context of ATL. Wong et al. performed enhancer profiling using primary leukemia samples from ATL, defined that the SEs at several known cancer gene loci, including *CCR4*, *PIK3R1*, and *TP73*, which are involved in the T-cell activation pathway in ATL. Notably, the study revealed that THZ1-mediated CDK7 inhibition ablates SE-driven oncogenic transcription in ATL, concomitantly suppressing proliferation and inducing apoptosis, underscoring SE-directed therapy as a viable treatment paradigm for this malignancy (Wong et al., 2017). Wong et al. also illustrated that IRF4 and NF-κB form a feed-forward regulatory loop that coordinately controls oncogenic transcription programs, with their binding sites being significantly enriched in SEs to directly modulate the expression of critical leukemia drivers including *MYC*, *CCR4*, and *BIRC3*, further promoting the ATL development (Wong et al., 2020). The HTLV-1-encoded bZIP gene (HBZ) is the sole viral transcription factor that continues to be expressed in all cases of ATL. Recent studies have demonstrated that HBZ binds to an ATL-cell–specific BATF3 SE, thereby regulating the transcription of the BATF3 factor and its downstream targets, including IRF4. BATF3 and IRF4 act synergistically to drive the expression of ATL-cell–specific genes and are essential for sustaining ATL cell proliferation. This HBZ-mediated SE activation allows the viral protein to directly perturb the host-cell gene-regulatory network, thereby promoting malignant proliferation of ATL cells (Nakagawa et al., 2018). SE-driven overexpression of *TP73*, a *TP53* homolog, promotes ATL maintenance by coordinately regulating cell cycle progression and DNA repair mechanisms (Ong et al., 2022). RUNX1 is a pivotal transcription factor in the development of hematopoiesis, and its dysregulation has been linked to various hematological malignancies. Research has indicated that the disruption of SE-mediated gene regulation can result in the suppression of RUNX1 expression, the induction of apoptosis, and the inhibition of ATL cell proliferation (Kobayashi et al., 2024). Collectively, these findings establish SEs as central epigenetic orchestrators of ATL pathogenesis, where they coordinate oncogenic transcription program, sustain proliferative signaling, highlighting the therapeutic potential of SE-directed interventions in ATL.

### 4.4 Dysregulated SEs in B-ALL and T-ALL pathogenesis

Acute lymphoblastic leukemia (ALL) is an aggressive hematological malignancy characterized by the uncontrolled proliferation of immature lymphoid precursors (B- or T-cell lineage) (Malard and Mohty, 2020). Recent advances highlight the critical role of SEs-mediated transcriptional dysregulation in ALL pathogenesis. In B-ALL, recurrent alterations targeting B-cell lineage transcription factors involve SE acquisition or disruption. Katerndahl et al. suggested a model in which the balance between STAT5 and a specific transcription factor network at SEs acts as a molecular switch to govern appropriate progenitor B cell proliferation, survival and differentiation (Katerndahl et al., 2017). Altering the balance between these two antagonistic pathways drives B cell transformation, while the degree of imbalance underlies how patients with B-ALL will respond to therapy. In addition, *PAX5* deletions or point mutations frequently co-occur with the formation of novel SEs at proto-oncogenes like MYC (Shah et al., 2013). Similarly, *IKZF1* (Ikaros) deletions or dominant-negative isoforms compromise its tumor-suppressive enhancer-silencing function, permitting SE-driven expression of cytokine receptors (*CRLF2*) and kinases (*JAK2*) (Raca et al., 2021; Mullighan, 2011).

SEs are crucial in regulating the expression of oncogenes in T-ALL. TAL1, one of the most frequently dysregulated genes in T-ALL is overexpressed in ∼50% of T-ALL cases, driven by mutations creating a 5′SE via MYB transcription factor binding (Smith et al., 2023). Noura et al. Have shown that KLF4 can downregulate *MYB* expression by directly binding to its promoter and inhibits the formation of 5′TAL1 SE, further suppresses SE-driven TAL1 expression in T-ALL cells (Noura et al., 2024). The *FYB1* gene driven by SEs is overexpressed in T-ALL and plays an important role in the self-renewal and survival of T-ALL cells. Knocking down *FYB1* leads to reduced tumor growth and increased cell apoptosis, highlighting its potential as a therapeutic target (Zhang et al., 2023). In addition, a recent study has shown that another SEs driven gene, *IRF2BP2*, is activated by specific SEs regions in T-ALL. This gene is crucial for the growth and survival of T-ALL cells, affecting pathways such as *MYC* and *E2F*, and *IRF2BP2* deficiency can impair the proliferation and survival of T-ALL cells (Yu et al., 2025). Targeting SEs and their related transcription mechanisms provides a promising therapeutic strategy in T-ALL. The BET inhibitor GNE-987 targets SEs related genes such as LCK, effectively inhibiting the progression of T-ALL (Yu et al., 2024). Understanding the mechanism of SEs formation and its related genes provides valuable insights into potential therapeutic targets and offers new avenues for treating T-ALL.

### 4.5 SEs driven oncogenic networks in MM

MM although a rare disease, is the second most common hematological malignancies. It is found in the spectrum of plasma cell dyscrasias, which begins with monoclonal gammopathy of unknown significance (MGUS) to overt plasma cell leukemia and extramedullary myeloma (Leung and Rajkumar, 2023). MM pathogenesis heavily relies on SE-driven transcription factor networks. MYC locus rearrangements, as a late progression event in MM, reposition *MYC* mostly with SEs which are associated with a significant increase of *MYC* expression (Affer et al., 2014). *HJURP* was identified as an SE-associated gene, which transcription is activated via chromatin interaction and binding of NSD2 and BRD4 to the SE associated with *HJURP.* SE, further promotes growth and survival of t (4; 14)-positive MM (Jia et al., 2022). As a member of the BET protein family, BRD4 recognises acetylated histones and promotes gene expression driven by SEs. BRD4 is highly enriched on SEs in MM. BET protein inhibitors can specifically displace BET proteins bound to SEs, leading to the potent, selective downregulation of SE target genes and the inhibition of MM cell proliferation (Fulciniti et al., 2015). SEs contribute significantly to drug resistance. Oncogenic overexpression of integrin-β7 (*ITGB7*) in high-risk MM has been reported to enhance interactions between neoplastic plasma cells and stromal cells, thereby promoting cell-adhesion-mediated drug resistance. Induction of DNA methylation at *ITGB7* SE further increases *ITGB7* expression promotes overall malignant growth in MM (Chou et al., 2023). MM is often driven by *MYC* and that is sustained by *IRF4*, which are upregulated by SEs. IKZF1 and IKZF3 bind to SEs and can be degraded using immunomodulatory imide drugs (IMiDs) (Welsh et al., 2024). The dependence of MM on IKZF1-bound SEs, which can be effectively targeted by a potent therapeutic combination pairing IMiD-mediated degradation of IKZF1 and IKZF3 with EP300 inhibition. Therefore, targeting SE components disrupts these resistance pathways and synergizes with conventional therapies.

**FIGURE 3:**
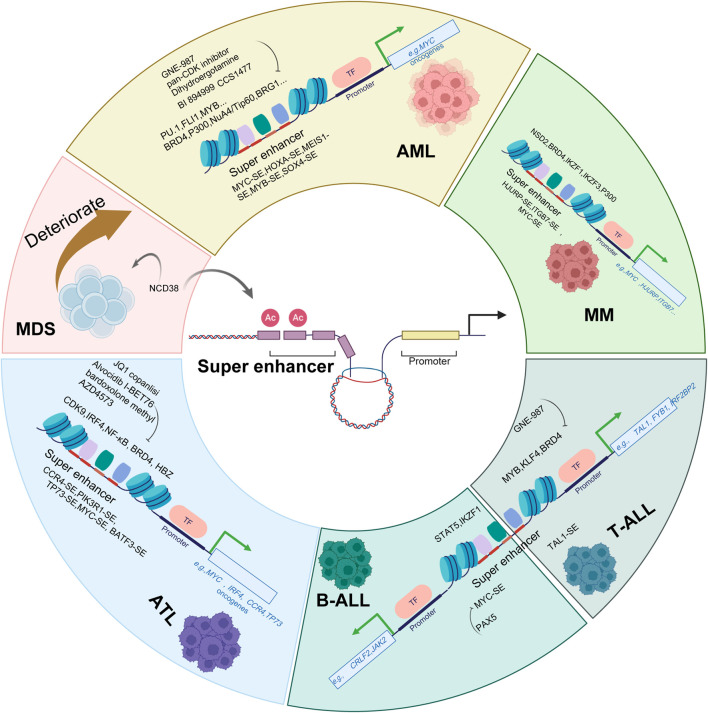
Targeting Super Enhancers: Therapeutic Strategies in Hematological Malignancies. Schematic diagram depicts the role of super enhancers (SEs) in driving hematological malignancies and the corresponding therapeutic strategies targeting SEs and their associated regulatory components. In various hematological malignancies, including acute myeloid leukemia (AML), adult T-cell leukemia/lymphoma (ATL), T-cell acute lymphoblastic leukemia (T-ALL), B-cell acute lymphoblastic leukemia (B-ALL), myelodysplastic neoplasms (MDS), and multiple myeloma (MM), aberrant SEs (e.g., MYC-SE, TAL1-SE, CCR4-SE, TP73-SE) drive oncogenic gene expression by recruiting transcription factors (e.g., PU.1, FLI1, MYB, STAT5, NF-κB) and coactivators (e.g., BRD4, p300, NuA4/Tip60 complex). Therapeutic agents such as GNE-987 (a BRD4 inhibitor), JQ1, pan-CDK inhibitors, alvocidib and AZD4573 (CDK9 inhibitor), bardoxolone-methyl (a NF-κB inhibitor), dihydroergotamine, BI894999 and I-BET76 (as BET inhibitor), CCS1477(a P300 inhibitor), copanlisi (a PI3K inhibitor), and NCD38 (an LSD1 inhibitor) disrupt SE-mediated oncogenic transcription by targeting key components (e.g., BRD4, CDK9, IRF4, NSD2, IKZF1/3, NF-κB) or directly interfering with SE function. These strategies aim to suppress the expression of critical oncogenes (e.g., MYC, TAL1, HOXA, IRF4) and thereby inhibit tumor progression in hematological malignancies.

In summary, SEs serve as pivotal drivers of oncogenic transcription across hematological malignancies (as shown in Figure 3). In AML, SEs are hijacked through various genetic alterations to activate key oncogenes such as *MYC*, *HOXA*, and *EVI1*, sustaining leukemogenic programs. In MDS, pathological silencing of SEs at hematopoietic differentiation genes contributes to disease pathogenesis, and pharmacological reactivation of these SEs represents a promising therapeutic strategy. Similarly, in ATL, SEs coordinate oncogenic transcription through feed-forward loops involving IRF4 and NF-κB, maintaining malignant phenotypes. In both B-cell and T-cell acute lymphoblastic leukemia (ALL), SEs dysregulation drives transformation by overexpressing oncogenes like *MYC* and *TAL1*, often through alterations in lineage-specific transcription factors. MM utilizes SE-driven networks centered on MYC and IRF4 to promote growth and drug resistance, with targeting of SE-associated complexes showing therapeutic potential. Collectively, these findings establish SEs as central epigenetic regulators in hematological malignancies and highlight SE-directed transcriptional inhibition as a viable therapeutic paradigm across cancer types.

## 5 SEs as promising therapeutic targets in hematological malignancies

SEs are potent transcriptional regulatory elements that drive the expression of genes fundamental to cell identity and survival. In hematological malignancies, the dysregulation or hijacking of SEs to activate oncogenes presents a compelling therapeutic vulnerability. Direct pharmacological targeting of the SE transcriptional machinery has emerged as a viable strategy and can be categorized by distinct mechanisms of action.

### 5.1 Targeting epigenetic readers and co-activators

A primary approach involves inhibiting bromodomain and extra-terminal (BET) proteins, which are critical for recognizing acetylated histones at SEs (Zhang et al., 2021). BET inhibitors, such as BI894999, have been shown to repress SE-associated transcription and control AML in preclinical models, often synergizing with CDK9 inhibition (Gerlach et al., 2018). Similarly, the BRD4 inhibitor GNE-987 exerts anticancer effects in AML by targeting a SE-related oncogen (Fang et al., 2022; Sang et al., 2022). Beyond BET proteins, inhibiting the EP300/CBP bromodomain (e.g., CCS1477 inhibitor) also represents a promising therapeutic avenue across various hematological malignancies (Nicosia et al., 2023). Additionally, JQ1, another BRD4 inhibitor, suppresses RUNX1 expression by disrupting SE-mediated gene regulation, further downregulating c-MYC and inducing apoptosis in ATL cells (Kobayashi et al., 2024).

### 5.2 Inhibiting transcriptional kinases

Targeting key transcriptional kinases such as CDK7 and CDK9, which facilitate RNA polymerase II‐mediated transcription at SEs, provides another therapeutic strategy. Small molecules co-targeting CKIα and CDK7/9 have shown efficacy in AML (Minzel et al., 2018). The CDK9 inhibitor alvocidib, for instance, suppresses ATL proliferation through SE-dependent downregulation of the oncogenic transcription factor IRF4 (Sakamoto et al., 2022).

### 5.3 Modulating oncogenic expression via SE interference

No-epigenetic pharmacological agents can also disrupt SE-driven oncogenic networks. Dihydroergotamine (DHE), for example, exhibits anti-AML activity by interfering with MYC expression through SE modulation (Call et al., 2020).

### 5.4 Rational combination therapies

The synergistic potential of SE-directed therapy is illustrated in ATL, where a triple combination of BET (I-BET762), PI3K (copanlisib), and NF-κB (bardoxolone methyl) inhibitors exhibits potent synergistic activity (Daenthanasanmak et al., 2022). Such combinations may enhance efficacy and mitigate resistance.

### 5.5 Resistance mechanisms and overcoming strategies

Despite promising results, therapeutic resistance remains a challenge. In multiple myeloma, transcriptional heterogeneity can enable escape from SE-targeting drug combinations (Welsh et al., 2024). However, targeting SE-driven dependencies can also overcome resistance, as demonstrated with the CDK9 inhibitor AZD4573, which induces epigenetic reprogramming to circumvent resistance in lymphoma models (Thieme et al., 2023).

In conclusion, the critical role of SEs in maintaining oncogenic programs positions them as attractive therapeutic targets. Targeting SE complexes and their co-factors, particularly through BET and CDK inhibitors, alone or in rational combinations, represents a promising direction for future cancer therapeutics in hematological malignancies.

## 6 Discussion and outlook

**FIGURE 4:**
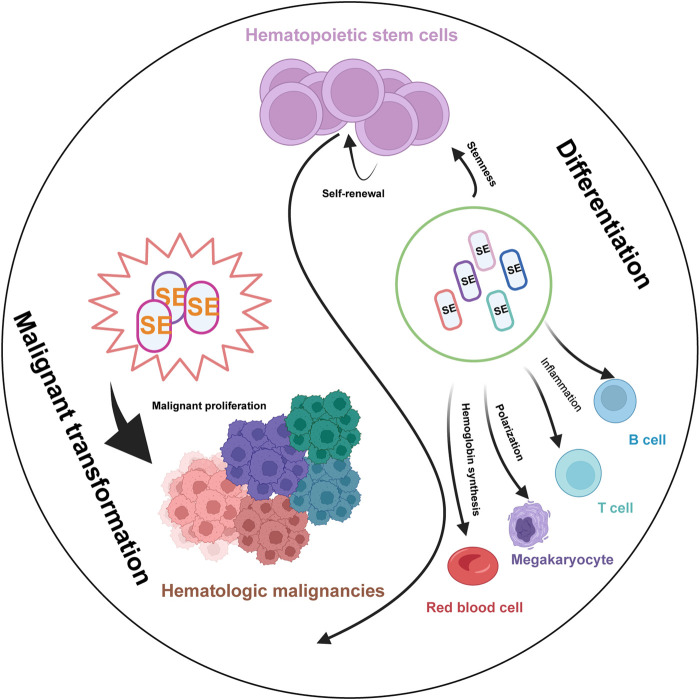
The Pivotal Role of SEs in Normal Hematopoiesis and Hematological Malignancies. Schematic illustrates the critical functions of SEs in regulating key biological processes during normal blood cell development (left) and their contribution to the pathogenesis of various hematologic malignancies (right). In normal hematopoiesis, Lineage-specific SEs (marks in different colors) govern fundamental processes such as stem cell self-renewal, lineage differentiation, and specialized functions including inflammation in immune cells and hemoglobin synthesis in erythroid cells. Following malignant transformation, dysregulated SEs drive oncogenic programs, leading to uncontrolled proliferation and the maintenance of the malignant state across different hematological malignancies.

SEs have emerged as pivotal regulators of gene expression in both normal hematopoiesis and hematological malignancies. Their unique ability to amplify transcriptional output through dense clustering of enhancer elements and coactivators underscores their critical role in maintaining cellular identity and driving oncogenic programs. As research progresses, the dual nature of SEs, as guardians of normal development and instigators of malignant transformation, has become increasingly apparent. As we have shown in Figure 4, SEs govern fundamental processes such as stem cell self-renewal and lineage differentiation in HSCs, and specialized functions including inflammation in immune cells and hemoglobin synthesis in erythroid cells, following malignant transformation, dysregulated SEs drive oncogenic programs, leading to uncontrolled proliferation and the maintenance of the malignant state across hematological malignancies.

In normal hematopoiesis, SEs function as master regulators of lineage-specific gene expression, ensuring the precise balance between self-renewal and differentiation of HSCs. For instance, SEs associated with MYC, RUNX1, and BRD4 are indispensable for HSCs maintenance and lineage commitment, as their disruption leads to aberrant differentiation and hematopoietic failure (Bahr et al., 2018; Liau et al., 2017). In macrophages, SEs dynamically regulate immune responses and inflammation related genes, which are essential for immune defense and maintaining inflammation homeostasis (Hah et al., 2015; Tanwar et al., 2025; Jia et al., 2021). Similarly, in erythroid cells, SEs not only regulate the differentiation but also orchestrate the spatiotemporal expression of globin genes (Xie and Dean, 2025; Alvarez-Dominguez et al., 2017; Hay et al., 2016; Kassouf et al., 2025; Gurumurthy et al., 2021), while in lymphoid lineages, they govern immune cell development and function through dynamic interactions with transcription factors like NF-κB and STAT5 (Michida et al., 2020; Wibisana et al., 2022; Zhou et al., 2015). The plasticity of SEs evidenced by their rapid remodeling in response to stimuli such as inflammation or infection, highlights their role as epigenetic hubs integrating environmental cues into transcriptional outputs.

In contrast, the hijacking of SEs in hematological malignancies exemplifies their pathogenic potential. By aberrantly activating oncogenes such as MYC, TAL1, and IRF4, SEs create a permissive environment for uncontrolled proliferation and survival of malignant clones. For example, in AML, chromosomal rearrangements reposition SEs to drive EVI1 or MYC overexpression (Roe et al., 2015; Gröschel et al., 2014; Ottema et al., 2021), while in T-ALL, SE-mediated TAL1 activation sustains leukemogenesis (Smith et al., 2023). Notably, SEs also contribute to therapeutic resistance, as seen in multiple myeloma, where SE-driven ITGB7 overexpression fosters cell-adhesion-mediated drug resistance (Chou et al., 2023). These findings underscore SEs as central nodes in the oncogenic networks of hematological cancers, often rendering tumors dependent on SE activity, a phenomenon termed “transcriptional addiction.”

The molecular characterization of SEs has spurred the development of targeted therapies aimed at disrupting their regulatory hubs. BET inhibitors (e.g., JQ1, GNE-987) and CDK inhibitors (e.g., THZ1, alvocidib) have shown preclinical efficacy by selectively downregulating SE-driven oncogenes (Wong et al., 2017; Kobayashi et al., 2024; Yu et al., 2024; Sakamoto et al., 2022; Fang et al., 2022). In AML and T-ALL, these agents induce apoptosis and differentiation by dismantling SE-associated transcriptional complexes. Similarly, LSD1 inhibitors (e.g., NCD38) reactivate silenced SEs to restore differentiation programs in MDS (Sugino et al., 2017). However, challenges remain, such as the fact that SEs regulate both oncogenic and normal hematopoietic genes, raising concerns about off-target effects; and the tumor cells may bypass SE dependency via compensatory mutations or alternative enhancer activation.

The convergence of multi-omics technologies and artificial intelligence is fundamentally reshaping our understanding of SEs in hematological malignancies. In diseases like AML and T-cell lymphoma, the integration of ChIP-seq with Hi-C has begun to precisely map the dynamic chromatin landscapes and three-dimensional interactions that underlie oncogenic SE hijacking (Hnisz et al., 2016; Koh et al., 2015). Crucially, single-cell multi-omics (e.g., scATAC-seq) is now unraveling the epigenetic heterogeneity of tumors, identifying distinct cellular subpopulations driven by unique SE programs that may confer therapy resistance (Satpathy et al., 2019). Complementing these approaches, deep learning models are being leveraged to predict functional enhancers directly from DNA sequence and to prioritize non-coding mutations that disrupt SE architecture. Together, these synergistic technologies are transitioning the field from descriptive mapping to the predictive modeling of SE networks, enabling the discovery of novel, context-specific therapeutic vulnerabilities directly from patient genomic data (Avsec et al., 2021).

SEs sit at the nexus of gene regulation, bridging normal development and malignant transformation. Their study has not only deepened our understanding of hematopoietic biology but also unveiled actionable targets for precision medicine. While challenges persist, the continued integration of epigenetic, genomic, and pharmacological approaches holds promise for transforming SE research into tangible clinical benefits, ultimately improving outcomes for patients with hematological malignancies.

## Glossary

**Aire**: Autoimmune regulator
**ALL**: Acute lymphoblastic leukemia
**AML**: Acute myeloid leukemia
**ATAC-seq**: Assay for Transposase-Accessible Chromatin using sequencing
**ATL**: Adult T-cell leukemia/lymphoma
**B-ALL**: B-cell acute lymphoblastic leukemia
**BET**: Bromodomain and extra-terminal domain
**BIRC3**: Baculoviral IAP repeat containing 3
**BRD4**: Bromodomain-containing protein 4
**C/EBPα**: CCAAT/enhancer binding protein alpha
**CBP**: CREB-binding protein
**CCR4**: C-C motif chemokine receptor 4
**CDK9**: Cyclin-dependent kinase 9
**CDKs**: Cyclin-dependent kinases
**ChIA-PET**: Chromatin Interaction Analysis by Paired-End Tag sequencing
**ChIP-seq**: Chromatin Immunoprecipitation sequencing
**Cpox-eRNA**: Coproporphyrinogen oxidase enhancer RNA
**CRLF2**: Cytokine receptor-like factor 2
**DHE**: Dihydroergotamine
**DNase-seq**: DNase I hypersensitive site sequencing
**E2F**: E2F transcription factor
**Epo**: Erythropoietin
**ERG**: V-ets erythroblastosis virus E26 oncogene homolog
**eRNAs**: Enhancer-derived RNAs
**FLI1**: Friend leukemia integration 1 transcription factor
**FYB1**: FYN binding protein 1
GATA2: GATA binding protein 2
**GFI1**: Growth factor independent 1 transcriptional repressor
**GNE-987**: A BRD4 inhibitor (no full name specified in manuscript)
**GRO-seq**: Global Run-On Sequencing
**H3K4me1**: Histone H3 lysine 4 mono-methylation
**H3K27ac**: Histone H3 lysine 27 acetylation
**Hi-C**: High-throughput Chromosome Conformation Capture
**HJURP**: Holliday junction recognition protein
**HOXA**: Homeobox A
**HOXA9**: Homeobox A9
**HSCs**: Hematopoietic stem cells
**HTLV-I**: Human T-cell leukemia virus type I
**IgH**: Immunoglobulin heavy chain
**IKZF1**: Ikaros family zinc finger 1
**IKZF3**: Ikaros family zinc finger 3
**IMiD**: Immunomodulatory imide drugs
**IRF2BP2**: Interferon regulatory factor 2 binding protein 2
**IRF4**: Interferon regulatory factor 4
**ITGB7**: Integrin beta 7
**JAK2**: Janus kinase 2
**LCK**: Lymphocyte-specific protein tyrosine kinase
**LDB1**: LIM domain binding 1
**LMO2**: LIM domain only 2
**lncRNAs**: Long non-coding RNAs
**LSD1**: Lysine-specific demethylase 1
**LYL1**: LYL1 basic helix-loop-helix transcription factor
**MDS**: Myelodysplastic neoplasms
**MED1**: Mediator complex subunit 1
**MEIS1**: Meis homeobox 1
**MGUS**: Monoclonal gammopathy of unknown significance
**MM**: Multiple myeloma
**MYB**: V-myb myeloblastosis viral oncogene homolog
**MYC**: myelocytomatosis oncogene
**NCD38**: A novel LSD1 inhibitor (no full name specified in manuscript)
**NFIX**: Nuclear factor I X
**NF-κB**: Nuclear factor kappa B
**NK cell**: Natural killer cell
**NSD2**: Nuclear receptor binding SET domain protein 2
**NuA4/Tip60**: Nucleosome acetyltransferase of H4/Tat-interactive protein 60 kDa
**p300/CBP**: E1A-binding protein p300/CREB-binding protein
**PAX5**: Paired box 5
**PIK3CD**: Phosphatidylinositol-4,5-bisphosphate 3-kinase catalytic subunit delta
**PIK3R1**: Phosphoinositide-3-kinase regulatory subunit 1
**PLAC-seq**: Proximity Ligation-Assisted ChIP-seq
**PRO-seq**: Precision Run-On Sequencing
**PU.1**: Putative oncogene PU.1
**RGL1**: Ral guanine nucleotide dissociation stimulator-like 1
**RNAP II**: RNA polymerase II
**RUNX1**: Runt-related transcription factor 1
**seRNAs**: Super enhancer-derived RNAs
**SEs**: Super enhancers
**SLC25A37**: Solute carrier family 25 member 37
**SLC4A1/BAND3**: Solute carrier family 4 member 1/Band 3
**SMARCA4**: SWI/SNF related, matrix associated, actin dependent regulator of chromatin, subfamily a, member 4
**SOX4**: SRY-box transcription factor 4
**SPNS2**: Spinster homolog 2
**STAT5**: Signal transducer and activator of transcription 5
**TAL1/SCL**: T-cell acute lymphocytic leukemia protein 1/stem cell leukemia
**T-ALL**: T-cell acute lymphoblastic leukemia
**THZ1**: A CDK7 inhibitor (no full name specified in manuscript)
**TLR4**: Toll-like receptor 4
**TP53**: Tumor protein p53
**TP73**: Tumor protein p73
**TRIB1**: Tribbles pseudokinase 1
**ZMYND11-MBTD1**: Zinc finger MYND-type containing 11-MBT domain containing 1 fusion protein
**ZMYND8**: Zinc finger MYND-type containing 8
